# Bibliometric analysis of research on digestive system tumors and depression

**DOI:** 10.3389/fpsyg.2024.1414528

**Published:** 2024-08-02

**Authors:** Ying Qu, Duorui Nie, Yuwei Song, Xiaojun Cai, Yilin Gong, Sheng Chen, Jia Ye, Jing Li

**Affiliations:** ^1^Department of Oncology, The First Hospital of Hunan University of Chinese Medicine, Changsha, China; ^2^Department of Oncology, Jiangsu Province Hospital of Chinese Medicine, Nanjing, China; ^3^School of Traditional Chinese Medicine, Hunan University of Chinese Medicine, Changsha, China; ^4^Department of Gynecology, The First Hospital of Hunan University of Chinese Medicine, Changsha, China

**Keywords:** digestive system tumors, depression, bibliometrics, visualization, Web of Science

## Abstract

**Background:**

Malignant tumors of the digestive system pose a serious threat to human health due to their highly malignant nature. Depression, as the most common psychiatric symptom of digestive system tumors, has attracted much attention regarding its potential relationship with these tumors. A thorough investigation into the connection between digestive system tumors and depression is extremely important for strengthening patients’ quality of life and treatment outcomes.

**Methods:**

From 2014 to 2023, we conducted a literature search using specific keywords in the Web of Science Core Collection (WoSCC) and performed visual analysis of the selected literature using Microsoft Excel, CiteSpace, and VOSviewer software. In this study, we analyzed countries, institutions, authors, journals, and keywords.

**Results:**

A total of 384 research articles on the relationship between digestive system tumors and depression were identified. The number of publications showed a gradual increase over time. In terms of disciplinary distribution, Oncology, Health Care Sciences Services, and Medicine General Internal ranked top in terms of publication volume. In terms of geographical distribution, China and the United States were the countries contributing the most publications. Additionally, Maastricht University contributed the most publications. Regarding authors, Beekman, Aartjan T.F. and Dekker, Joost had the highest number of publications, while Zigmond, A.S. had the most citations. It is worth mentioning that *Supportive Care in Cancer* was the journal with the most publications in this field. In terms of keyword analysis, research mainly focused on mechanisms and treatment strategies related to the relationship between digestive system tumors and depression.

**Conclusion:**

The relationship between digestive system tumors and depression has become a new research hotspot in recent years, offering new directions for future research. This research reveals novel perspectives on comprehending the connection between the two, which can guide future research and practice.

## 1 Introduction

Depression is a prevalent mental health issue that is defined by enduring melancholy, diminished interest and enjoyment, impaired focus and cognitive abilities, which severely affect the quality of life and social functioning of patients (Freeman, 2022). According to World Health Organization data, there are over 300 million people globally suffering from depression, and this number continues to rise (Friedrich, 2017). Depression not only causes significant psychological stress to individuals but also imposes a serious burden on society and the economy. It is projected that by 2030, depression will become the leading cause of the global disease burden (Reddy, 2012). Assessing depression from the perspective of bodily self is an indispensable approach. Bodily self refers to an individual’s subjective experience and perception of their physical appearance and function, playing a central role in shaping one’s self-image and mental health (Sebri et al., 2023). Negative bodily self often leads to decreased self-esteem and a diminished sense of self-worth, thereby increasing the risk of depression (Gallese and Ferri, 2014).

One of the most prevalent psychological symptoms among cancer patients is depression (Zabora et al., 2001). There exists a complex and close relationship between depression and tumors (Nelson et al., 2010). Studies have shown that cancer patients are more likely to experience depressive emotions, with a prevalence of emotional disorders in the tumor population approaching 40% (Krebber et al., 2014). Patients with cancer may have a shorter survival time and a lower quality of life when depressed (Smith, 2015; Wang et al., 2020). Preventing and treating the comorbidity of tumors and depression can slow down tumor progression, prolong patient survival (Kissane, 2014), and provide new insights into the prevention and treatment of tumors (Grassi and Riba, 2020).

Digestive system malignant tumors account for approximately 50% of all malignant tumors (Yuan Yuan et al., 2022), mainly including esophageal cancer, gastric cancer, colorectal cancer, pancreatic cancer, hepatocellular carcinoma, and biliary tract cancer, which are the most common causes of cancer-related deaths worldwide (Xi and Xu, 2021). Especially among patients with digestive system tumors, the incidence of depressive emotions is relatively high (Mussell et al., 2008), and it significantly impacts the recovery and treatment outcomes of patients. For patients with digestive system tumors, the physical changes brought about by the tumor and its treatment often have a profound impact on their body self-representation (Laricchiuta et al., 2023). Digestive system tumors and their treatment can lead to significant weight changes, surgical scars, ostomies, and prolonged periods of physical discomfort and pain. These physical changes can affect patients’ satisfaction with their appearance and increase emotional distress, thereby triggering negative emotions and depressive symptoms (González et al., 2016).

As far as we know, current research on the relationship between tumors and depression largely focuses on breast cancer and lung cancer (Pössel et al., 2012), while more comprehensive study and exploration is needed, the basic and clinical research on depression and digestive tract malignancies is now insufficient (Trudel-Fitzgerald et al., 2022).

Bibliometrics is a field that scrutinizes scientific literature to discern the development trends, academic influence, hot topics, and related relationships in academic research areas (Ninkov et al., 2022). It does so by examining various indicators like quantity, quality, citation relationships, author collaborations, journals, and keywords, all of which offer insights into how the field is evolving. In this regard, conducting a bibliometric analysis on studies that investigate the link between digestive system tumor and depression would be of great theoretical and practical importance. With this in mind, our goal is to use bibliometric and visualization methods to uncover the publication and citation patterns of various countries, institutions, journals, authors, and hot topics in the field. We aim to gain a better understanding of the historical research landscape, current research directions, and to make predictions about future developments in this area.

## 2 Methods

### 2.1 Data collection

We have chosen to utilize the Web of Science Core Collection (WoSCC) as the primary source for literature retrieval. WoSCC is considered the most important global citation database, containing a vast number of academic articles from renowned journals across multiple disciplines, including open-access journals, conference proceedings, and books. We have searched WoSCC for all articles related to digestive system tumor and depression from 2014 to 2023 using the following search formula: TS = (cecal OR duodenal OR ileal OR jejunal OR esophageal OR oesophagus OR esophagus OR gastrointestinal OR gastric OR intestinal OR pancreatic OR colorectal OR colon OR stomach OR rectum OR anus OR rectal OR liver OR gallbladder OR Hepatic OR Gastrointestinal) AND (Cancer OR carcinoma OR malignancies OR Malignant OR Malignancy OR Neoplasia OR Neoplasm OR tumour OR tumor) AND (depression OR “depressive disorder” OR “depressive symptom”).

### 2.2 Inclusion criteria

The inclusion criteria for the literature review are as follows: (1) English-language literature; (2) focusing on the correlation between digestive system tumors and depression; (3) including basic research, clinical studies, and reviews related to digestive system tumors and depression.

### 2.3 Exclusion criteria

The exclusion criteria for the literature review are as follows: (1) non-English-language literature; (2) book chapters, proceedings papers, conference abstracts, editorial materials, online publications, errata, data papers, letters, news, retractions, etc.; (3) irrelevant articles. Initially, a total of 3,156 papers were obtained, which reduced to 2,746 papers after selecting articles and reviews from 2014 to 2023. After selecting English-language articles, the final count reduced to 2,706 papers.

### 2.4 Analysis methods and tools

Using Microsoft Excel to draw charts based on the literature retrieved from WoSCC database, including trend charts for the number of publications and citations, analyzing the distribution of disciplines related to digestive system tumors and depression, and ranking countries, institutions, authors, and journals.

CiteSpace is a tool used for visualizing and analyzing citation networks of academic literature (Synnestvedt et al., 2005). It can generate various visualizations such as time-series graphs, co-citation networks, keyword co-occurrence maps, etc. By analyzing citation relationships and keyword co-occurrence in literature, CiteSpace reveals the evolution process and key nodes in research fields, providing powerful visual support for academic research. We have exported all data retrieved from WoSCC in plain text format and import it into CiteSpace V6.2.R6 analysis software. Parameters are set to include the time slice from 2014 to 2023, with each slice representing 1 year. In this study, the software is mainly used for keyword co-occurrence analysis.

VOSviewer is mainly used for analyzing and visualizing keyword co-occurrence networks and author collaboration networks in academic literature (van Eck and Waltman, 2010). VOSviewer can transform literature data into network graphs and maps, revealing key research topics, academic collaboration relationships, and interdisciplinary information in research fields through analysis of keywords, authors, journals, etc. The software is mainly used in this study for the following tasks: (i) analysis of country citations; (ii) analysis of author collaboration and co-citations; (iv) analysis of journal citations and co-citations; and (v) analysis of keyword co-occurrences.

## 3 Result

### 3.1 Publication outputs and citation frequency

**Figure 1:**
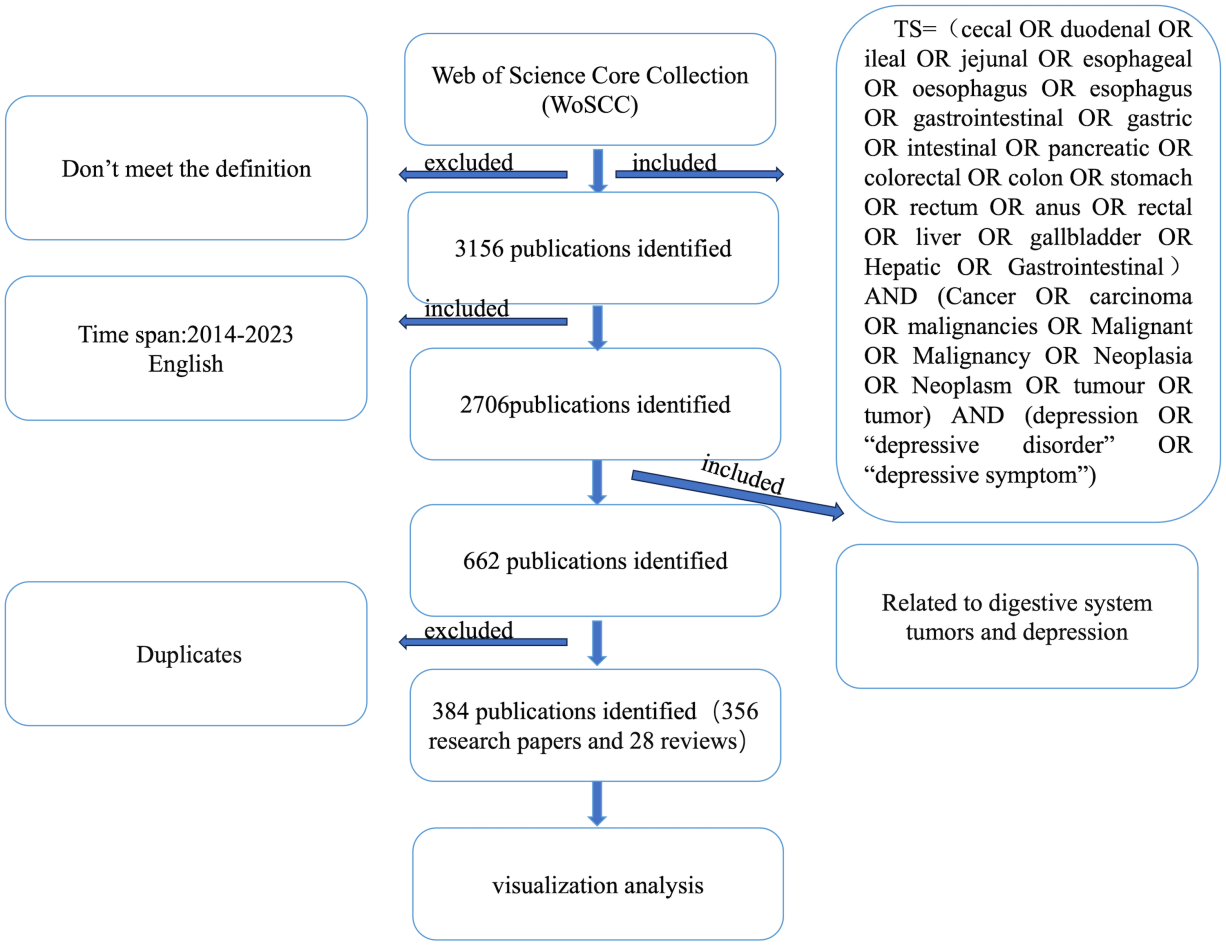
PRISMA flowchart.

**Figure 2:**
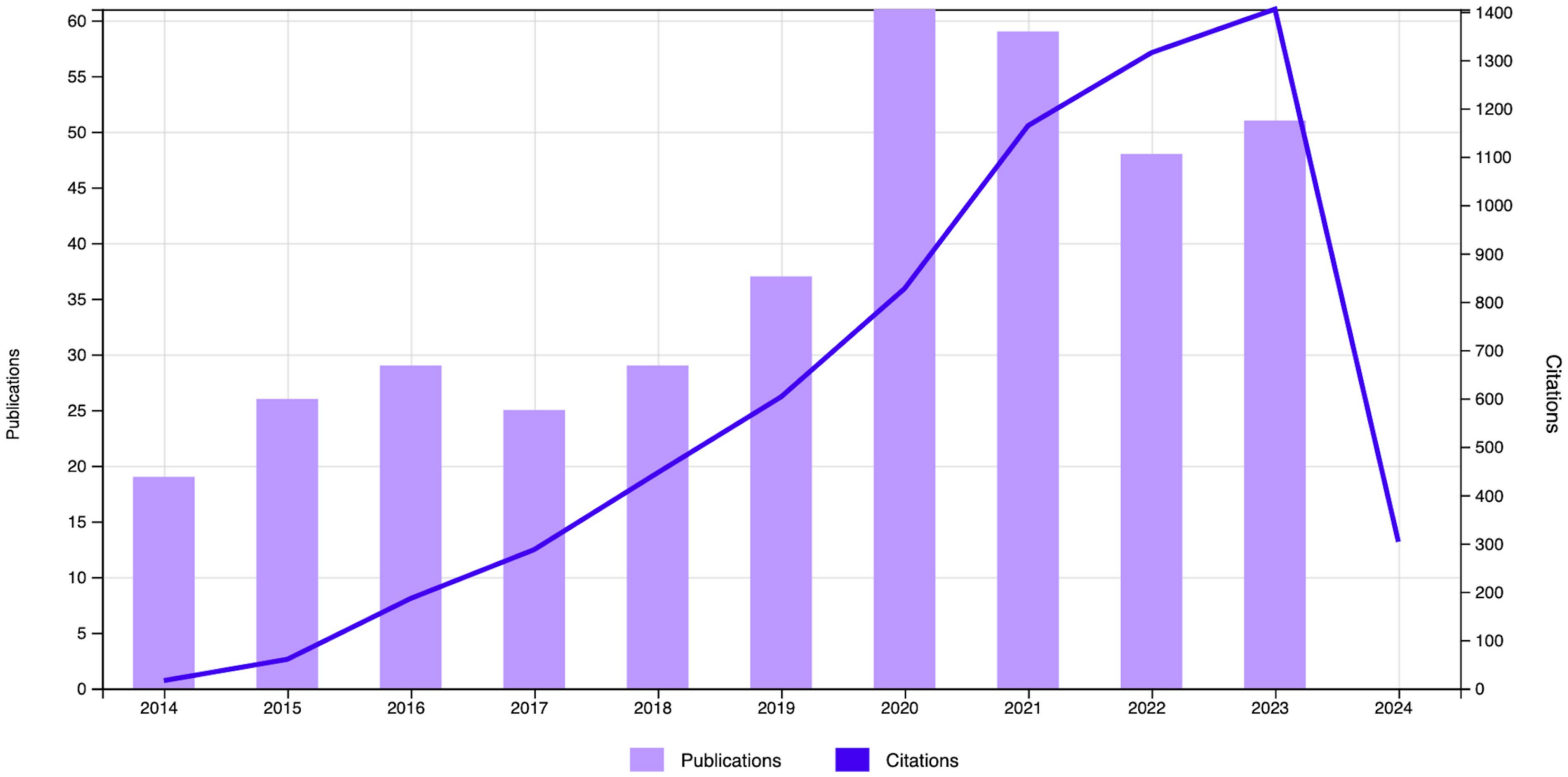
Annual trends in publication and citation.

From our retrieval strategy, a total of 662 articles related to digestive system tumors and depression were published from 2014 to 2023. After removing duplicates using CiteSpace, 384 articles remained, including 356 research papers and 28 reviews (Figure 1). As shown in Figure 2, the trend of publication output indicates an upward trend in the past 10 years regarding the topic of digestive system tumors and depression. From 2014 to 2016, the number of articles published annually increased steadily. Although the growth rate slowed slightly between 2016 and 2019, the overall trend still rised. The number of articles published increased significantly between 2020 and 2022, with a noteworthy growth rate, particularly in 2020 and 2021. However, there was a slight decrease in the number of articles published from 2022 to 2023. In terms of citation frequency, there was a significant increase in the number of citations each year from 2014 to 2023. Although the growth rates varied between some years, overall, there was a consistent upward trend, indicating a certain level of stability. This steady growth over the long term may reflect the ongoing attention and involvement in the relationship between digestive system tumors and depression.

### 3.2 Subject categories of Web of Science

**Figure 3:**
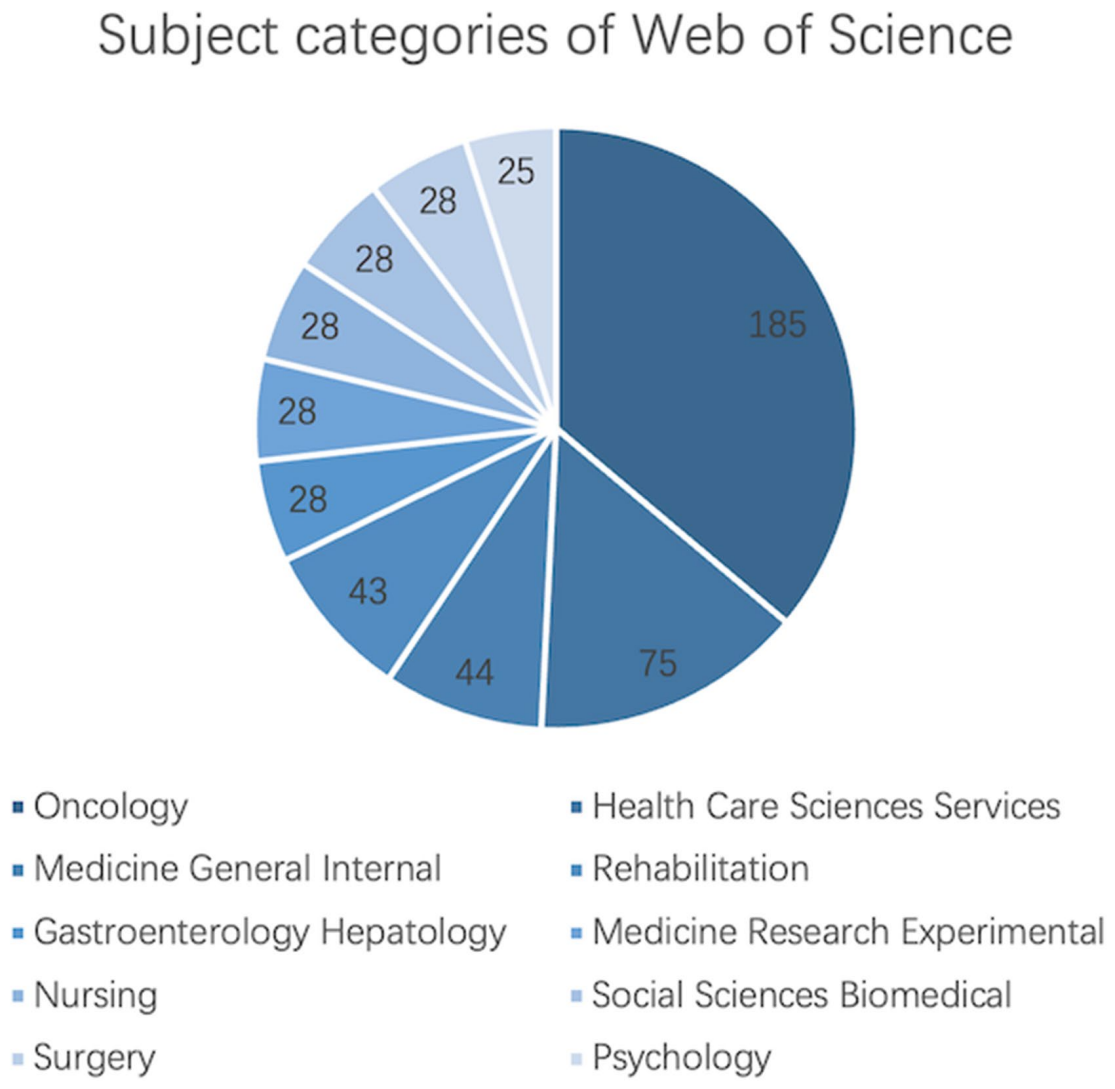
Digestive system tumors and depression rank among the top 10 subjects.

Among all literature related to digestive system tumors and depression, we analyzed the top 10 subjects based on the number of published articles (Figure 3).

Oncology had the highest number of published papers, with a total of 185 articles. The top five subjects also included Health Care Sciences Services, Medicine General Internal, Rehabilitation, and Gastroenterology Hepatology. Psychology had the lowest number of publications, with only 25 articles. Subjects with higher publication rates may have some correlation or interdisciplinary research, such as Oncology and Gastroenterology Hepatology possibly having intersecting research in the treatment of certain types of tumors. Despite having lower publication rates, Psychology, as a social science, also contributes to research and knowledge in the medical and health fields. Overall, this data highlights the differences in publication rates across various subjects, reflecting their level of activity and influence in academic research. This kind of analysis assists in comprehending the academic research aims and trending subjects, guiding decisions on academic investment and research directions.

### 3.3 Contributions of countries

**Figure 4:**
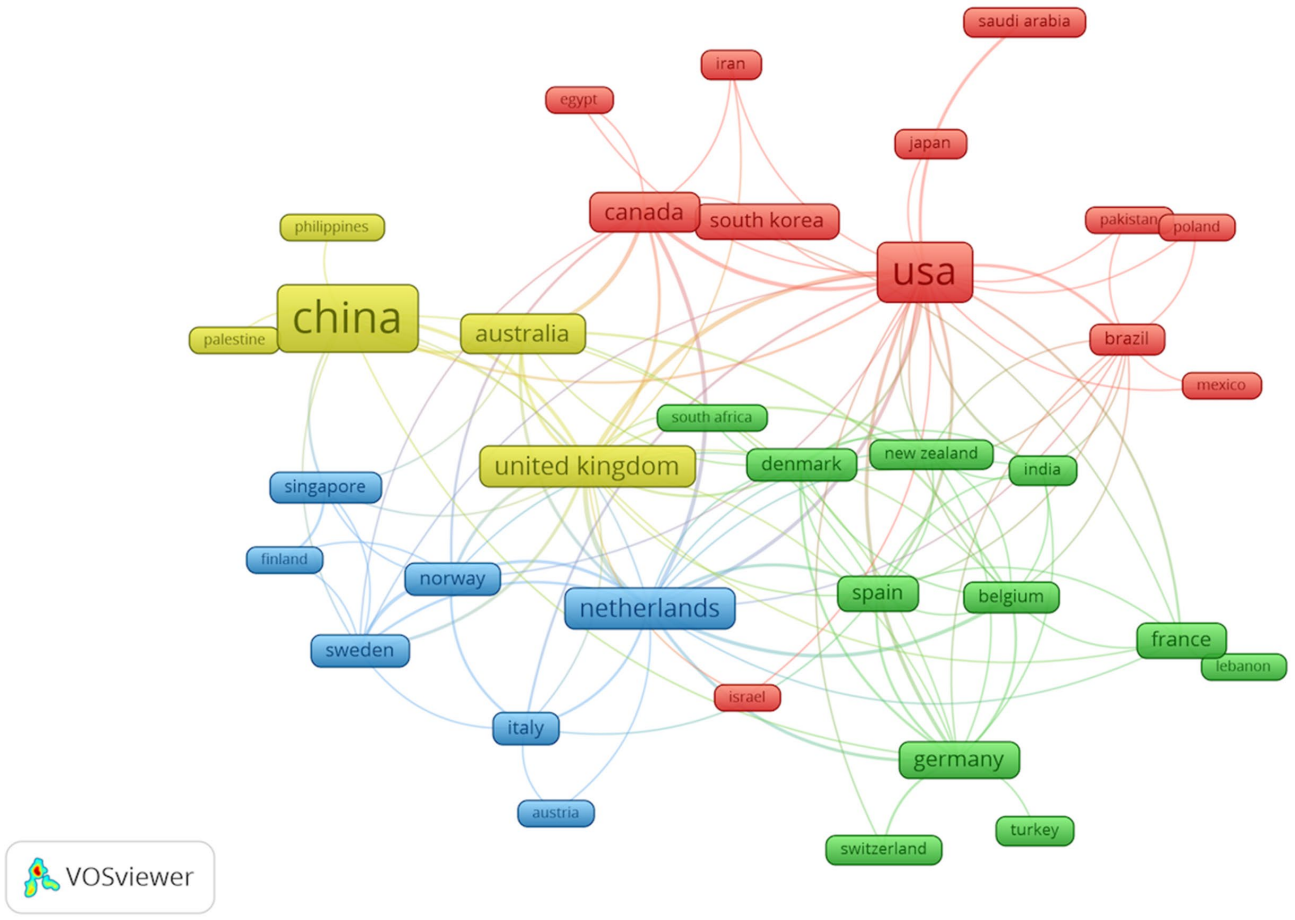
Publication countries visualization.

As Table 1 shows, China and the United States are the top two countries in terms of publication output related to the relationship between digestive system tumors and depression, with 112 and 90 publications, respectively. These two countries are highly active in research in this field and demonstrate a high level of research expertise and productivity. European countries such as the United Kingdom, the Netherlands, Germany, Spain, and France also have a considerable number of publications in this field, with 32, 29, 21, 18, and 16 publications, respectively. These countries also contribute significantly to research on the relationship between digestive system tumors and depression. Countries like Australia, Canada, and South Korea have relatively fewer research activities in this field, with 27, 25, and 17 publications, respectively. Subsequently, we selected and visualized 35 countries and constructed a collaboration network based on the publication volume and relationships of each country (Figure 4).

**Table 1:** The top 10 countries in terms of publication output on digestive system tumors and depression.

Rank	Countries	Counts	Percent
1	China	112	29.16%
2	USA	90	23.44%
3	UK	32	8.33%
4	Netherlands	29	7.55%
5	Australia	27	7.03%
6 7 8 9	Canada Germany Spain Korea	25 21 18 17	6.51% 5.47% 4.69% 4.43%
10	France	16	4.17%

### 3.4 Contributions of institutions

Nine hundred and fifty-four institutions in all have contributed to publications about the connection between digestive system tumors and depression. Among the top 10 research institutions in terms of publication output (Table 2), the majority are from the United States and the Netherlands. Maastricht University contributed the most publications, followed by the University of Toronto and Dana-Farber Cancer Institute. The top three institutions with the highest citation counts are University Medical Center Utrecht (360 citations), University of Toronto (334 citations), and Maastricht University (182 citations).

**Table 2:** The top 10 institutions in terms of publication output on digestive system tumors and depression.

Rank	Institutions	Countries	Counts	TLS	Total citations
1	Maastricht univ	Netherlands	13	19	182
2	univ Toronto	Canada	10	15	334
3	Dana farber canc inst	USA	7	14	104
4	univ calif san francisco	USA	9	14	113
5	univ med ctr utrecht	Netherlands	6	14	360
6	univ amsterdam	Netherlands	6	13	56
7	vrije univ. Amsterdam	Netherlands	6	13	88
8	harvard med sch	USA	8	9	84
9	mayo clin	USA	5	9	123
10	nci	USA	5	9	68

**Figure 5:**
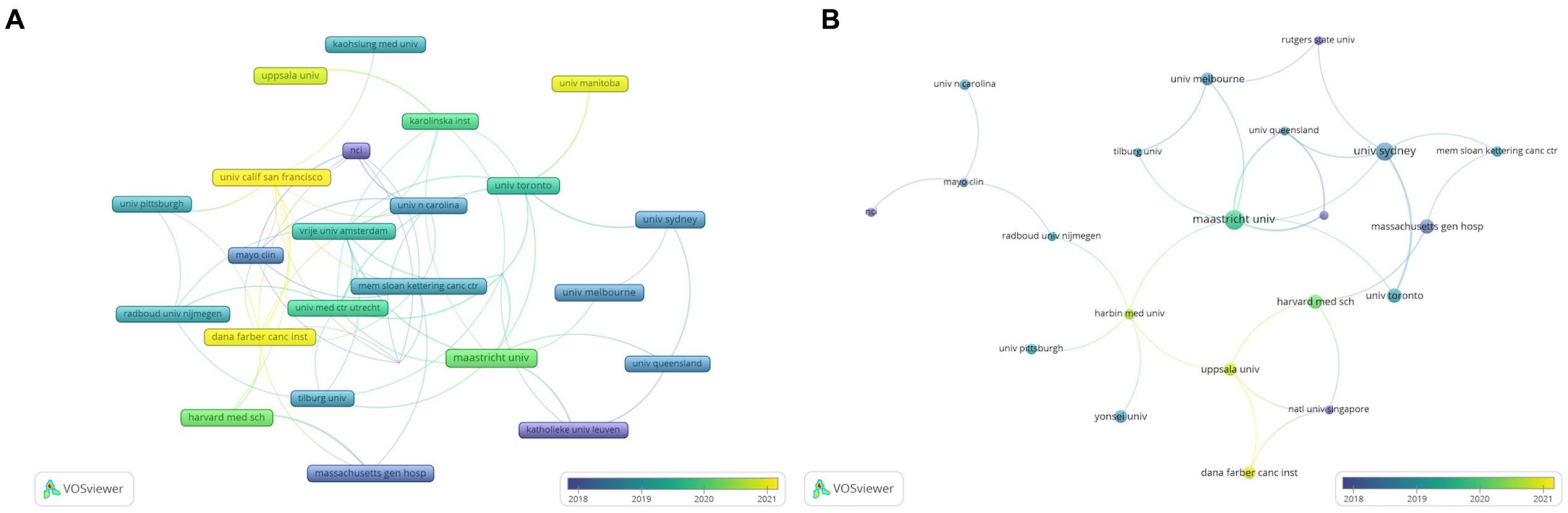
Publication institutions visualization. **(A)** VOSviewer overlay visualization map of the institutions’ citation; **(B)** VOSviewer overlay visualization map of the institutions’ co-authorship.

VOSviewer generated a network visualization map for institution citation and co-authorship analysis. As shown in the overlaid visualization map (Figure 5A), institutions are color-coded based on their Average Authorship Year (AAY) values. According to the color gradient displayed in the bottom right corner, some institutions like Massachusetts General Hospital, NCI, and Katholieke Universiteit Leuven are assigned larger purple AAY values, indicating that most researchers from these institutions were early contributors to the field and had a greater impact on the research of the relationship between digestive system tumors and depression.

Conversely, a lot of the institutions highlighted in yellow might be relatively recent additions to this research, represented by institutions like the University of California San Francisco and Dana-Farber Cancer Institute. As shown in Figure 5B, the co-authorship visualization map highlights institutions with higher recent citation rates in green and deep yellow, while purple represents institutions with higher citation rates in earlier years. Higher TLS values indicate greater impact, with larger nodes displayed in the co-authorship visualization map for publishing institutions. The top three institutions in terms of overall impact based on TLS are as follows: Maastricht University, University of Toronto, and Dana-Farber Cancer Institute.

### 3.5 Authors and co-cited authors

**Figure 6:**
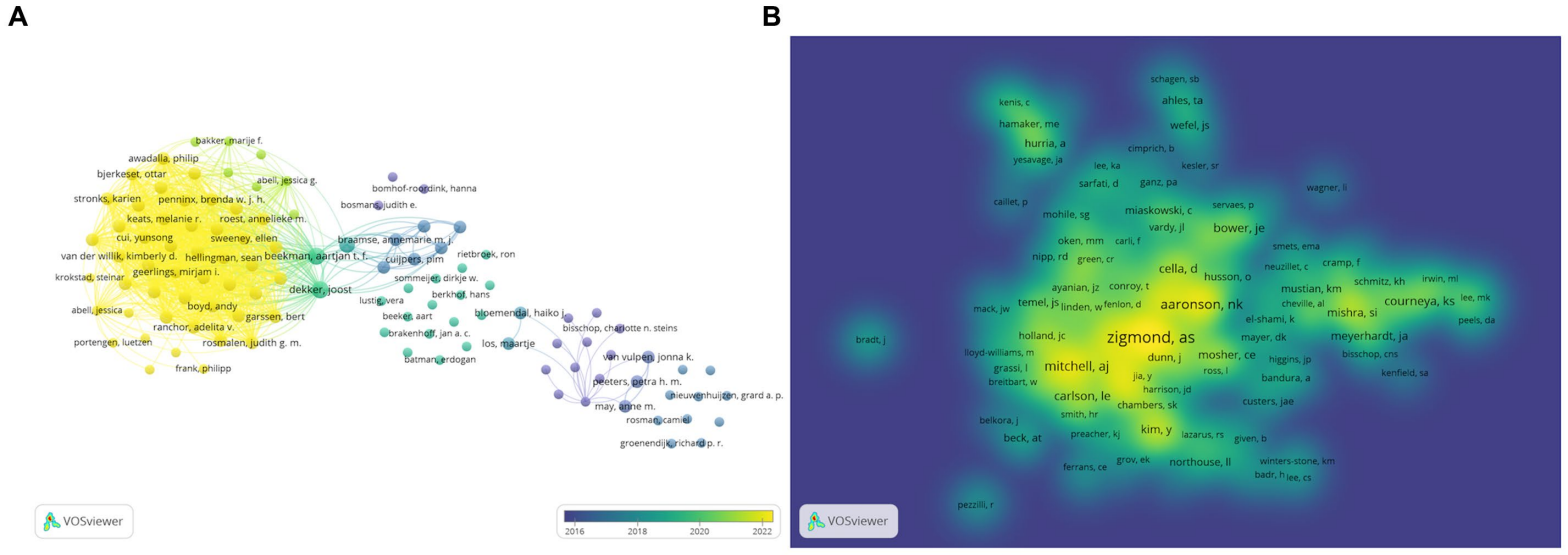
Publication authors visualization. **(A)** VOSviewer overlay visualization map of the authors; **(B)** VOSviewer density visualization map of the authors.

A total of 9,428 authors have participated in research on the relationship between digestive system tumors and depression. Among the top 10 authors listed in (Table 3), two authors each have published four papers. We established a collaboration network graph based on authors who have published two or more papers (Figure 6A). The nodes for Beekman, Aartjan T.F. and Dekker, Joost are the largest because they have published the most relevant publications and have closer collaboration with other authors. The majority of nodes in the graph are shown in yellow, indicating an increase in research on the relationship between digestive system tumors and depression in recent years. Furthermore, we analyzed the relationship graph between co-cited authors (Figure 6B). The author’s citation count increases with node size and color brightness. With 86 citations, As Zigmond has the most of all of them, followed by NK Aaronson with 53 citations, and AJ Mitchell with 37 citations.

**Table 3:** The top 10 authors in terms of publication output on digestive system tumors and depression.

Rank	Authors	Counts	TLS	Total citations
1	Beekman, aartjan.t.f	4	34	99
2	Dekker, joost	4	34	99
3	Awadalla, philip	2	5	81
4	Basten, maartje	2	5	81
5	Bjerkeset, ottar	2	5	81
6	Boyd, andy	2	5	81
7	Cui, yunsong	2	5	81
8	De graeff, alexander	2	5	81
9	Galenkamp, henrike	2	5	81
10	Garssen, bert	2	5	81

### 3.6 Journals and co-cited journals

In this research field, a total of 168 journals have appeared. As shown in (Table 4), *Supportive Care in Cancer* has published the most papers, followed by *Psycho-oncology* with 21 papers. The journal *Cancer* has the highest impact factor of 6.2, followed by *Journal of Cancer Survivorship* with an impact factor of 3.7. Among the top 10 journals, six are from the United States, two are from the United Kingdom, one is from Germany, and one is from Scotland. Based on the analysis of the Journal Citation Indicator (JCI), research on digestive system tumors and depression has a high citation impact in the fields of oncology and gastroenterology, indicating the importance and influence of these studies in these areas. In the field of psychiatry, although the JCI is relatively low, the significance of the research remains notable. This could be because cross-disciplinary studies in this field are relatively scarce, making the impact of each individual study more critical.

**Table 4:** The top 10 journals in terms of publication output on digestive system tumors and depression.

Rank	Journals	Countries	Counts	IF (5 Year)	Total citations	JCR	JCI (2023)
1	Supportive care in cancer	Germany	39	3.2	746	Q2	0.96
2	Psycho-oncology	UK	21	4.4	429	Q1	0.8
3	International journal of clinic and experimental medicine	USA	12	0.2	4	Q4	0.02
4	European journal of oncology nursing	UK	11	0.315	127	Q2	0.98
5	Journal of pain and symptom management	USA	10	3.8	138	Q1	1.3
6	cancer	USA	9	6.7	526	Q1	1.29
7	American journal of translation research	USA	7	2.3	29	Q3	0.44
8	Bmj open	UK	7	2.7	101	Q2	0.68
9	Journal of cancer survivorship	USA	7	4.1	372	Q2	0.81
10	medicine	USA	7	1.6	51	Q3	0.35

**Figure 7:**
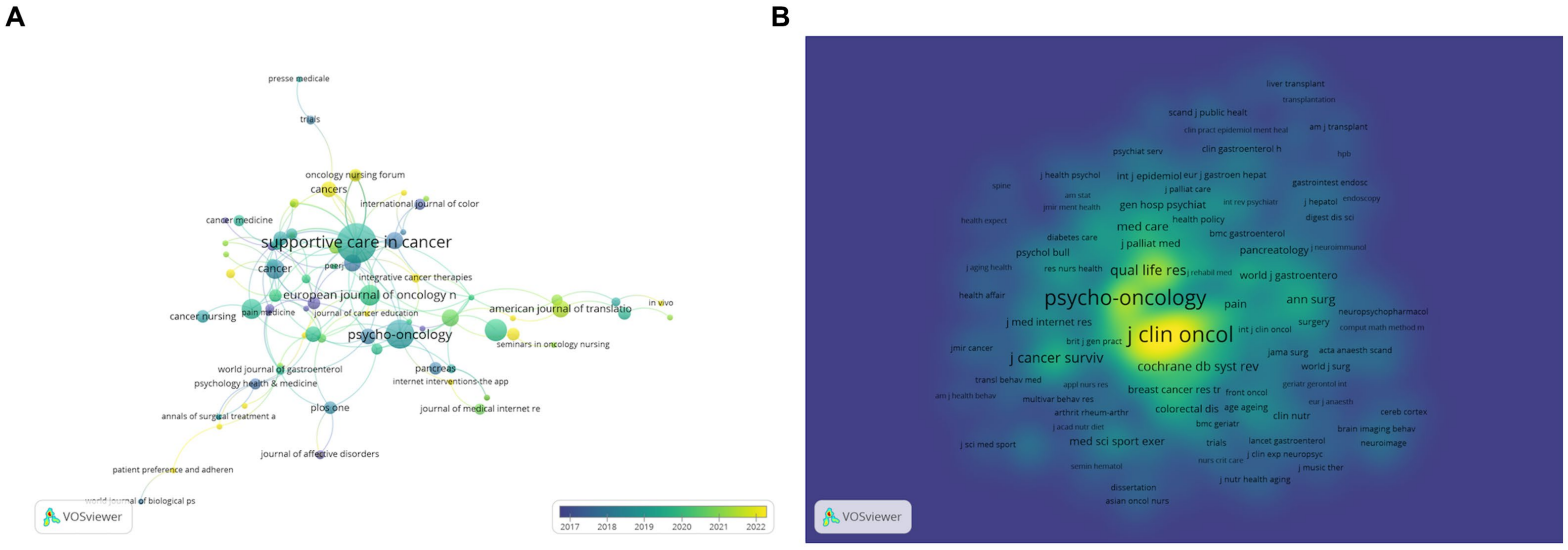
Publication journals visualization. **(A)** VOSviewer overlay visualization map of the journals; **(B)** VOSviewer density visualization map of the journals.

The journal citation map (Figure 7A) shows that *Supportive Care in Cancer* has the largest and darkest node, indicating that this journal has published many papers in this field and has been publishing for a long time. The top 3 journals with the highest total link strength (TLS) are *Supportive Care in Cancer* (TLS = 38), *Journal of Cancer Survivorship* (TLS = 11), and *Psycho-oncology* (TLS = 9). The co-citation journal map (Figure 7B) shows 469 nodes and 37,425 connections. In terms of influence, in the field of the relationship between digestive system tumors and depression, journals such as *J Clin Oncol*, *Psycho-oncology*, and *J Cancer Surviv* are very active, with a high co-citation rate. This indicates that the publications from these journals have higher quality than average.

### 3.7 Keywords analysis of research hotspots

**Figure 8:**
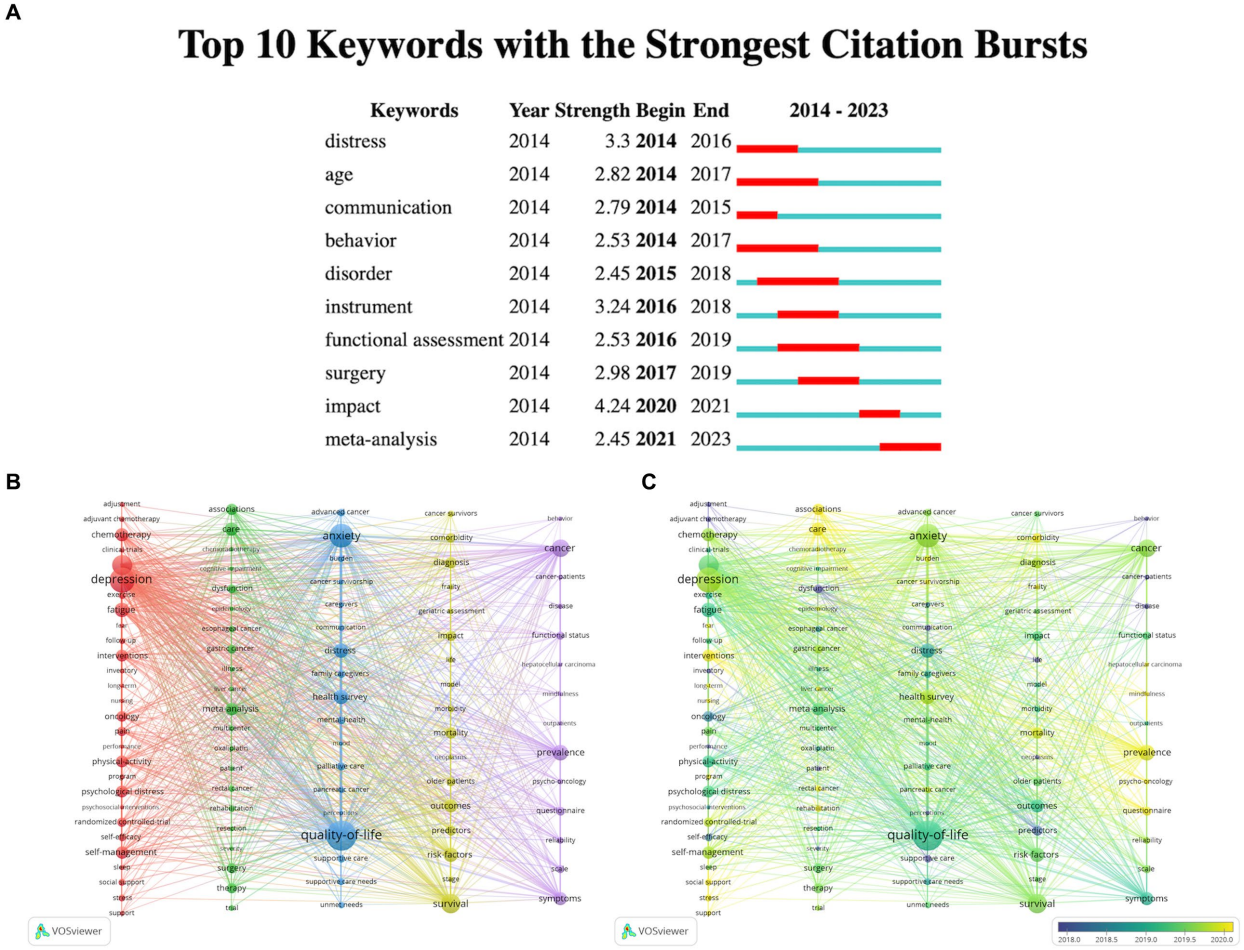
Publication keywords visualization. **(A)** Top 10 keywords in the strongest citation burst. **(B)** VOSviewer overlay visualization map of the keywords; **(C)** VOSviewer density visualization map of the keywords.

“Keyword burst” refers to keywords that are frequently cited within a certain period, and they can be used to predict research frontiers based on the distribution of the strongest keyword bursts. We analyzed the keywords related to the topic of digestive system tumors and depression using CiteSpace (Figure 8A). Keywords such as “age,” “behavior,” “disorder” and “functional assessment” have a long duration of bursts.

We imported publications related to digestive system tumors and depression from WoSCC into VOSviewer software, selecting author keywords and keywords plus, and divided these keywords into five clusters represented by different colors (Figure 8B). The larger the node, the more frequent the keyword appears, indicating its importance in the topic of the relationship between digestive system tumors and depression. Among them, “quality-of-life,” “depression,” “anxiety,” “distress” and “cancer” are the most frequently appearing keywords, reflecting their importance and level of attention in the related literature.

We divided the keywords into 5 clusters and extracted high-frequency keywords from each cluster as follows: In Cluster 1, the frequently occurring keywords are “depression,” “fatigue,” “interventions,” “physical activity” and “self-management.” In Cluster 2, the frequently occurring keywords are “care,” “surgery,” “therapy” and “dysfunction.” In Cluster 3, the frequently occurring keywords are “quality-of-life,” “anxiety,” “distress” and “health survey.” In Cluster 4, the frequently occurring keywords are “survival,” “risk factors,” “diagnosis,” “impact,” “outcomes” and “predictors.” In Cluster 5, the frequently occurring keywords are “cancer,” “prevalence” and “symptoms.”

The overlay visualization map (Figure 8C) shows that green keywords indicate an earlier average appearance year compared to other colors, while yellow-colored keywords indicate a later average appearance year. “Quality-of-life” and “distress” have deeper colors, indicating their earlier appearance and reflecting researchers’ early attention to the quality of life and psychological distress of cancer patients. “Depression,” “anxiety” and “cancer” have lighter colors, indicating their relatively later appearance and reflecting the shifting focus of researchers in this field towards mental health issues and their association with cancer.

## 4 Discussion

In discussing the research results on the comorbidity of digestive system tumors and depression, we employed bibliometric methods to analyze literature in the related field. Rational use of CiteSpace and VOSviewer tools for statistical and visualization analysis was adopted, with the aim of grasping the research dynamics and trends in this field, thereby providing guidance for future studies. The analysis results reveal that the overall number of publications has been on a growing trend, especially from 2019 to 2021, which may be related to the increased focus on depression research during the COVID-19 pandemic (Daly and Robinson, 2022; Peng et al., 2022). The characteristics of interdisciplinary research are significant, involving fields such as medicine, nursing, and social sciences, among which oncology, healthcare science services, and internal medicine are the disciplines with the highest level of attention.

Through international collaborative analysis, it has been found that China and the United States play a central role in this field of research, signaling an intensification of international cooperation. In terms of institutions, Maastricht University in the Netherlands and the University of Toronto have the highest publication output, while the Netherlands and the United States are the main national contributors. Citation analysis reveals that while some institutions, like the University Medical Center Utrecht in the Netherlands, may not have a high volume of publications, they have a significant number of citations, indicating their substantial influence in the field. Well-known institutions such as Harvard Medical School, the National Cancer Institute (NCI), and the Mayo Clinic also hold positions in the rankings.

Author collaboration and citation analysis reveal that Beekman, Aartjan T.F. and Dekker, Joost are among the leaders in terms of publication output and collaborative relationships, while Zigmond, AS is the most cited author. He is a British psychiatrist, and the Hospital Anxiety and Depression Scale (HADS), which he developed in 1983, is primarily used for screening anxiety and depressive moods in general hospital patients (Zigmond and Snaith, 1983). In terms of journals, American journals keep a prominent position of leadership in this field. The impact factors and citation numbers of the Journal of *Pain and Symptom Management* and *Cancer* are particularly prominent, indicating their high academic status and influence in this research field (Scott et al., 2024).

The distribution of research topics in the field of digestive system tumors and depression from 2014 to 2023 is shown by a keyword co-occurrence analysis. We are able to reveal the frontiers and hotspots of this field’s research when we combine it with overlay visualization maps. The results show that research on the digestive system tumors and depression is mainly focused on mechanisms and treatment. At the same time, we have also noted that depression is a neurological disease, and the onset of digestive system tumors is related to the digestive system. After searching for more related literature, we find that an increasing number of researchers are focusing on the relationship between tumors and depression in the gut microbiota. The microbiota-gut-brain (MGB) axis has many paths via which the gut bacteria might impact the brain (Ortega et al., 2022). The dysbiosis of the gut microbiota not only disrupts the micro-ecological balance of the human body but also increases the risk of digestive system tumors (Alpert et al., 2021). A case–control study found that the intestinal microbial characteristics of patients with both digestive system tumors and depression differ from other populations (Zhu et al., 2021). We will next explore in detail the mechanisms of the microbiota-gut-brain axis in digestive system tumors and depression, as well as possible treatment options based on these mechanisms.

In recent years, the association between the gut microbiota and digestive system tumors has attracted widespread attention. The gut is not only the site where a large number of immune cells are produced (Mou et al., 2022), but the balance of its microbiota is crucial for human health. Studies have pointed out that when this microbiota is imbalanced, it may lead to increased permeability of the intestinal epithelial barrier, and subsequently the release of pro-inflammatory factors (Collins et al., 2012), such as TNF-α, IL-1β, and IL-6. These factors not only exacerbate intestinal inflammation but may also trigger neuroinflammation in the body, promoting the occurrence and metastasis of tumors (Djaldetti and Bessler, 2014). In addition, we have found that the level of inflammation in patients with depression is also significantly increased (Skowrońska et al., 2023), which has been confirmed in both human and animal model studies of depression (Chen et al., 2024; Chu et al., 2024). Inflammatory factors have become a major biomarker for digestive system tumors and depression. We speculate that the specific mechanism may be that on one hand, inflammatory factors cause endothelial cells to contract, reducing tight junction proteins, thereby making the blood–brain barrier more permeable (Mayhan, 2001); on the other hand, inflammatory factors activate brain immune cells, such as microglia, leading to neuroinflammation, which is crucial for brain development. Additionally, these factors activate NF-κB, AP-1, Raf, and MAPK-mediated signaling pathways, further influencing tumor growth and metastasis (Sahin and Aricioglu, 2013).

Within the neuroendocrine system, the hypothalamic–pituitary–adrenal (HPA) axis is a crucial component. Dysfunctions in the HPA axis, particularly overactivation, can lead to increases in corticotropin-releasing factor (CRF), adrenocorticotropic hormone (ACTH), and cortisol (CORT). This is not only closely related to the onset of depression but also affects the incidence of cancer (Oh et al., 2019; Abate et al., 2020). Patients with digestive system tumors often face intense anxiety regarding their prognosis, potential side effects of treatment, and the impact of the disease on their daily lives. This psychological stress can trigger a stress response characterized by elevated cortisol levels (Naser et al., 2021). Overactivation of cortisol can suppress immune function, reduce the body’s immune defense and immune surveillance against tumor cells, thereby contributing to the growth and incidence of tumors (Yee and Li, 2021). At the same time, anxiety about treatment outcomes and fear of recurrence can lead to chronic stress, further impairing immune function and overall health. Additionally, changes in the gut microbiota can also affect the activity of the HPA axis through brain-gut axis feedback signals, thereby affecting emotional states (Silva et al., 2020; Liu et al., 2021). Metabolic products produced by the gut microbiota, such as short-chain fatty acids (SCFA), can be transported to the brain via the bloodstream, influencing related neural pathways and neurotransmitter systems in the brain, leading to depression (O'Riordan et al., 2022). These interactions highlight the bridging role of the brain-gut axis between depression and digestive system tumors.

In the study of comorbidity between digestive system tumors and depression, in addition to the currently analyzed keywords, omics research is also an emerging research trend. Particularly, research on the tryptophan metabolism pathway provides new insights and potential biomarkers.

Tryptophan is an essential amino acid and a precursor to 5-hydroxytryptamine (5-HT). Tryptophan can be metabolized through two main pathways: the 5-HT pathway and the kynurenine (KYN) pathway (Davidson et al., 2022). In a healthy state, tryptophan is metabolized by tryptophan hydroxylase (TPH) and 5-hydroxytryptamine decarboxylase (AADC) into 5-HT, which is an important neurotransmitter involved in regulating mood, sleep, and appetite (McLean et al., 2007). The expression of 5-HT has also been found in various digestive system tumors, promoting tumor occurrence and development (Ye et al., 2021).

In inflammatory or tumor environments, inflammatory cytokines such as IFN-γ, IL-6, and TNF-α can increase the activity of indoleamine 2,3-dioxygenase (IDO) (Xu et al., 2022). The increased IDO activity causes more tryptophan to be metabolized through the kynurenine pathway rather than the 5-HT pathway, reducing the synthesis of 5-HT. Reduced levels of 5-HT are associated with depressive symptoms (Pan et al., 2019; Deng et al., 2021).

At the same time, increased IDO activity converts tryptophan into KYN (Chen et al., 2021). KYN is a metabolite that can cross the blood–brain barrier and is further metabolized in the central nervous system into several different products, including the neurotoxic quinolinic acid (QUIN) (Lin et al., 2023). QUIN can activate N-methyl-D-aspartate (NMDA) receptors, leading to neuronal damage and death (Huang et al., 2020).

Therefore, in inflammatory or tumor environments, increased IDO activity not only reduces the synthesis of 5-HT, leading to depressive symptoms, but also increases levels of KYN and its neurotoxic metabolites (such as QUIN), negatively affecting the nervous system. This metabolic imbalance is closely related to the occurrence and development of depression and also plays an important role in the pathological process of digestive system tumors.

Increasing evidence indicates that antidepressants not only act by modulating neurotransmitters to affect depression but also possess immunosuppressive and anti-inflammatory properties (Schmidt et al., 2016). A meta-analysis has demonstrated that anti-inflammatory treatment, particularly with selective COX-2 inhibitors such as celecoxib, can effectively reduce depressive symptoms (Köhler et al., 2014). Additionally, glutamate modulators, like ceftriaxone, can alleviate depression symptoms by inhibiting neuroinflammation via the GLT-1/TrkB signaling pathway (Gao et al., 2024). The dual action of antidepressants thereby improves mental health on one hand, and on the other, can suppress the release of inflammatory factors, combating the development and progression of tumors.

Controlling the balance of the gut microbiota by fecal microbiota transplantation or probiotic usage, can help improve symptoms of depression and may also have a positive effect on the treatment of digestive system tumors. Fecal microbiota transplantation (FMT) is a treatment method that introduces the fecal microbiota from a healthy person into a patient’s body (Chen et al., 2019). It can reduce intestinal inflammation, rebuild the gut microbiota, and optimize the intestinal immune microenvironment, thereby achieving the purpose of treating the comorbidity of digestive system tumors and depression (Antushevich, 2020). Probiotics, as supplements, can inhibit neuroinflammation and protect the blood–brain barrier by reducing the release of cortisol and pro-inflammatory cytokines, promoting the health of brain function and neural activity (Cai et al., 2023). Comprehensive studies show that probiotic supplements have a significant therapeutic effect on improving gut function and depressive states (Moya-Pérez et al., 2017; Kim et al., 2021; Paiva et al., 2024).

Psychotherapy can help patients effectively deal with depressive emotions, thereby improving their quality of life and psychological health status (Lauer, 2015). In addition, nursing and social support also play a crucial role (Gonzalez-Saenz de Tejada et al., 2017; Zhang et al., 2020), providing comprehensive care services and social support networks to help patients better cope with the challenges of the disease, and enhance their confidence and willpower to fight the disease. Furthermore, interventions such as Mindfulness-Based Cognitive Therapy (MBCT), Acceptance and Commitment Therapy (ACT), and Mindfulness-Based Stress Reduction (MBSR) are currently commonly used for patients with digestive system tumors who also suffer from depression (Nissen et al., 2020; Xunlin et al., 2020). Studies have shown that these psychological interventions can effectively reduce the overactivation of the HPA axis, alleviate emotional stress, and thus reduce intestinal inflammation through the brain-gut axis, affecting the progression of digestive system tumors.

In summary, current research has revealed to us the comorbidity relationship between digestive system tumors and depression, but there are still certain limitations. Although the concept of the brain-gut axis provides us with a theoretical framework, the exploration of key biomarkers, molecular pathways, and cellular mechanisms is far from sufficient. In the future, we still need to continue our exploration in hopes of finding new potential therapeutic targets, providing more effective references and guidelines for clinical practice. Through research on the theme of comorbidity of digestive system tumors and depression with CiteSpace and VOSviewer, a deep understanding of the current research status and development trends in this field can provide guidance and suggestions for future research directions.

### 4.1 Strengths and limitations

It’s impressive that the research is pioneering in the field of digestive system tumors and depression from a bibliometric perspective. Using Microsoft Excel, CiteSpace, VOSviewer, and other visualization tools demonstrates a comprehensive approach to analyzing research trends and collaboration networks. However, it’s important to acknowledge the limitations, such as relying solely on the WoSCC database and focusing exclusively on English-language articles. Excluding non-English literature and gray literature might result in incomplete research findings. On the one hand, bibliometric analysis focuses on the dissemination and impact of research results, while neglecting the research process itself, such as methodological innovations, data collection, and analysis procedures. On the other hand, it primarily emphasizes quantitative metrics like the number of publications and citation counts, while overlooking the content quality of the literature. Additionally, the manual selection of articles related to the topic by researchers may introduce selection bias.

## 5 Conclusion

This article summarizes the literature on digestive system tumors and depression extracted from WoSCC from 2014 to 2023. Through visual analysis of the data, we observed an increasing trend in both the number of publications and citation frequency related to digestive system tumors and depression. China and the United States are the top contributors in terms of publication volume. We believe that future research should focus more on keyword-based analysis. Through bibliometric analysis, researchers can better understand the academic dynamics of comorbid digestive system tumors and depression, thereby identifying research priorities and conducting more in-depth and systematic studies to explore the biological mechanisms and pathological connections between the two. Uncovering key research hotspots and future development trends can provide guidance for clinicians and health policymakers, enabling them to incorporate mental health interventions into comprehensive treatment strategies, thereby improving the overall health and quality of life of patients.

## Data availability statement

The datasets presented in this study can be found in online repositories. The names of the repository/repositories and accession number(s) can be found at: Web of Science.

## Author contributions

YQ: Formal analysis, Software, Visualization, Writing – original draft. DN: Formal analysis, Writing – original draft, Writing – review & editing. YS: Data curation, Writing – review & editing. XC: Data curation, Writing – review & editing. YG: Data curation, Writing – review & editing. SC: Software, Writing – review & editing. JY: Software, Visualization, Writing – review & editing. JL: Conceptualization, Data curation, Funding acquisition, Supervision, Writing – review & editing.
