# Corrigendum: Bibliometric analysis of research on digestive system tumors and depression

**DOI:** 10.3389/fpsyg.2024.1497693

**Published:** 2024-12-20

**Authors:** Ying Qu, Duorui Nie, Yuwei Song, Xiaojun Cai, Yilin Gong, Sheng Chen, Jia Ye, Jing Li

**Affiliations:** ^1^Department of Oncology, The First Hospital of Hunan University of Chinese Medicine, Changsha, China; ^2^Department of Oncology, Jiangsu Province Hospital of Chinese Medicine, Nanjing, China; ^3^School of Traditional Chinese Medicine, Hunan University of Chinese Medicine, Changsha, China; ^4^Department of Gynecology, The First Hospital of Hunan University of Chinese Medicine, Changsha, China

**Keywords:** digestive system tumors, depression, bibliometrics, visualization, Web of Science

In the published article, there were errors in the affiliations (1, 2, 4). Instead of “1 Department of Oncology, The First Affiliated Hospital of Hunan University of Chinese Medicine, Changsha, China”, “2 Graduate School, Hunan University of Chinese Medicine, Changsha, China”, and “4 Department of Gynecology, The First Affiliated Hospital of Hunan University of Chinese Medicine, Changsha, China”, it should be “1 Department of Oncology, The First Hospital of Hunan University of Chinese Medicine, Changsha, China”, “2 Department of Oncology, Jiangsu Province Hospital of Chinese Medicine, Nanjing, China”, and “4 Department of Gynecology, The First Hospital of Hunan University of Chinese Medicine, Changsha, China”.

Additionally, in the published article, there was an error in Figure 1. Two corrections have been made. Firstly, an error in the literature source has been rectified to ensure that all included documents are in English. Secondly, the oversight in the literature count has been corrected by properly summing the number of articles and reviews. The corrected Figure 1 and its caption appear below.

**Figure 1:**
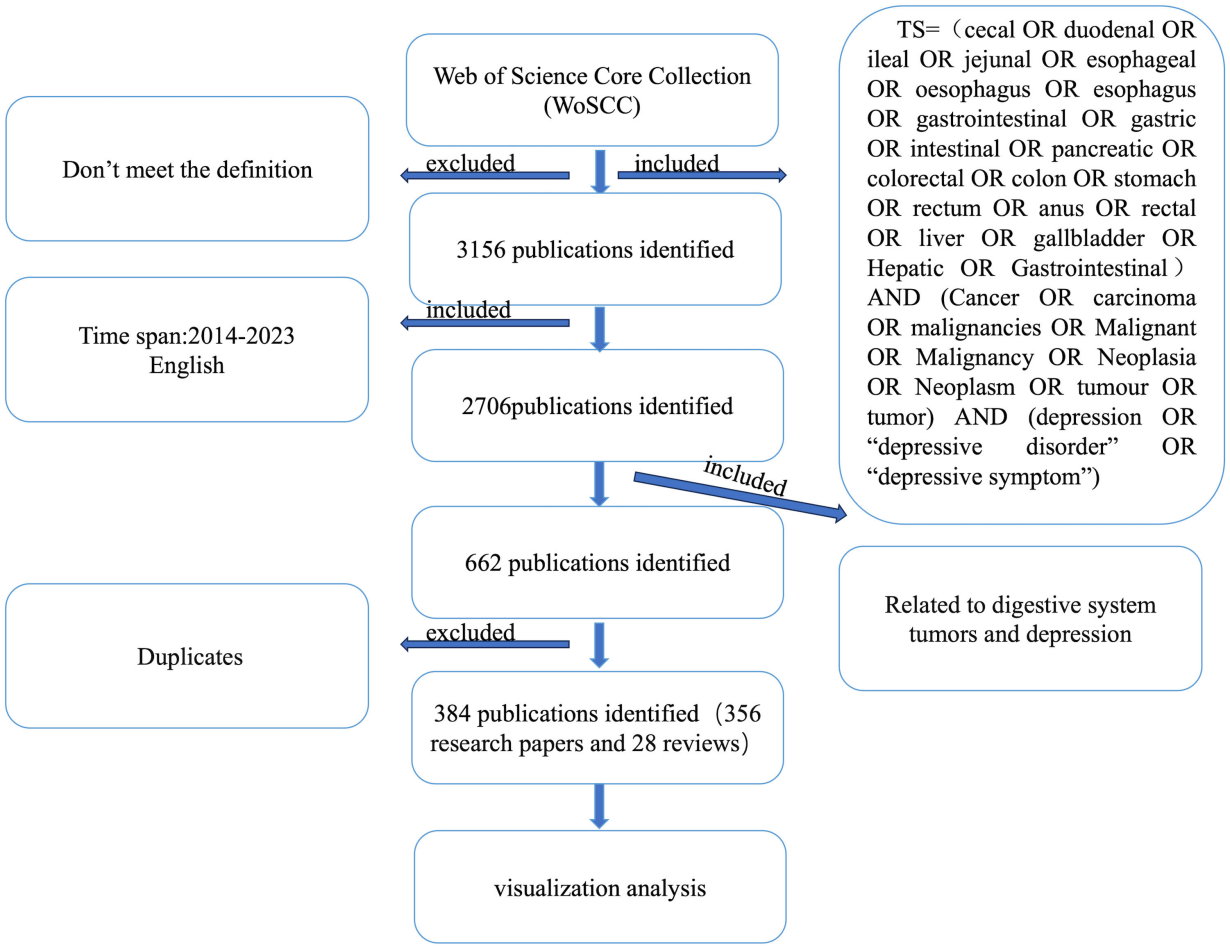
PRISMA flowchart.

Lastly, in the published article, there was an error in the Funding statement. The authors failed to list all the funding project numbers completely and omitted the name of an important funding project. The correct Funding statement appears below.

